# Readiness to deliver person‐focused care in a fragile situation: the case of Mental Health Services in Lebanon

**DOI:** 10.1186/s13033-021-00446-2

**Published:** 2021-03-02

**Authors:** Aya Noubani, Karin Diaconu, Giulia Loffreda, Shadi Saleh

**Affiliations:** 1 Global Health Institute, American University of Beirut, Beirut, Lebanon; 2 NIHR Research Unit on Health in Situations of Fragility, Institute for Global Health and Development, Queen Margaret University, Musselburgh, UK; 3 Department of Health Management and Policy, Faculty of Health Sciences, American University of Beirut, Beirut, Lebanon

**Keywords:** Mental health, Health system, Primary care, Healthcare providers, Fragility, Lebanon, Community members

## Abstract

**Background:**

Evidence suggests wide variability in the provision of mental healthcare across countries. Countries experiencing fragility related risks suffer from a high burden of mental-ill health and additionally have limited capacity to scale up mental health services given financial and human resource shortages. Integration of mental health services into routine primary care is one potential strategy for enhancing service availability, however little is known about the experiences of currently active health care providers involved in mental health and psychosocial support service (MHPSS) provision at primary care level. This study aims to determine how healthcare providers offering MHPSS services at primary care levels in Lebanon perceive mental health and the health system’s ability to address the rising mental ill-health burden with a view to identify opportunities for strengthening MHPSS service implementation geared towards integrated person focused care model.

**Methods:**

A qualitative study design was adopted including 15 semi-structured interviews and 2 participatory group model-building workshops with health care providers (HCPs) involved in mental healthcare delivery at primary care level. Participants were recruited from two contrasting fragility contexts (Beirut and Beqaa). During workshops, causal loop diagrams depicting shared understandings of factors leading to stress and mental ill health, associated health seeking behaviors, and challenges and barriers within the health system were elicited. This research is part of a larger study focused on understanding the dynamics shaping mental health perceptions and health seeking behaviours among community members residing in Lebanon.

**Results:**

Findings are organized around a causal loop diagram depicting three central dynamics as described by workshop participants. First, participants linked financial constraints at household levels and the inability to secure one’s livelihood with contextual socio-political stressors, principally referring to integration challenges between host communities and Syrian refugees. In a second dynamic, participants linked exposure to war, conflict and displacement to the occurrence of traumatic events and high levels of distress as well as tense family and community relations. Finally, participants described a third dynamic linking cultural norms and patriarchal systems to exposure to violence and intergenerational trauma among Lebanon’s populations. When describing help-seeking pathways, participants noted the strong influence of social stigma within both the community and among health professionals; the latter was noted to negatively affect patient-provider relationships. Participants additionally spoke of difficulties in the delivery of mental health services and linked this to the design of the health system itself, noting the current system being geared towards patient centered care, which focuses on the patient’s experiences with a disease only, rather than person focused care where providers and patients acknowledge broader structural and social influences on health and work together to reach appropriate decisions for tackling health and other social needs. Barriers to delivery of person focused care include the lack of coherent mental health information systems, limited human capacity to deliver MHPSS services among primary health care staff and inadequate service integration and coordination among the many providers of mental health services in our study contexts. Critically however, provider accounts demonstrate readiness and willingness of health professionals to engage with integrated person focused care models of care.

**Conclusions:**

Mental ill health is a major public health problem with implications for individual health and wellbeing; in a fragile context such as Lebanon, the burden of mental ill health is expected to rise and this presents substantive challenges for the existing health system. Concrete multi-sectoral efforts and investments are required to (1) reduce stigma and improve public perceptions surrounding mental ill health and associated needs for care seeking and (2) promote the implementation of integrated person focused care for addressing mental health.

**Supplementary Information:**

The online version contains supplementary material available at 10.1186/s13033-021-00446-2.

## Introduction

Mental ill health is one of the greatest global health challenges of this decade, affecting a broad range of individuals across all age groups [1]. Estimates show that mental health problems account for 32.4 % of years lived with disability and 13% of disability adjusted life years (DALY) globally [2]. Over 80 % of persons affected by mental health and psychosocial issues reside in low and middle income countries (LMICs) [3]. This is largely due to communities in these countries being affected by man-made and natural disasters, including conflict and war, earthquakes and epidemics. All of the latter are found to increase the incidence of mental health problems, while simultaneously also eroding the capacity of communities and health systems to respond to this and other health issues [3]. Other factors, such as poverty, urbanization, lifestyle changes and stigma were also found to contribute to the increased burden of mental health issues in LMIC populations [3].

Despite the considerable burden of mental health issues and associated adverse human, economic and social effects, care for persons experiencing mental health and psychosocial challenges remains limited in LMICs [1]. Findings from the World Mental Health Surveys show that 76–85 % of people with serious mental health problems receive no treatment for their disorder in LMICs compared to 35–50 % of those in high income countries [4]. This wide variability in persons receiving mental health treatment as well as health systems providing mental health care is a result of several challenges. First, financing of mental health services remains inadequate, especially in LMICs where less than 1 % of health budgets is allocated to addressing mental illness on average [5]. Investments in the mental health workforce, both in terms of recruitment and training, remain limited due to competing public health priorities in these settings. Second, limited coordination remains a key barrier to easy access and comprehensive, integrated care delivery. The majority of LMIC health systems report having no dedicated information system for capturing and reporting basic mental health (MH) needs; hence, health systems are also unable to coordinate the delivery of services and assess the magnitude of the mental ill-health burden [3, 6]. Lack of information and coordination additionally implies limited coordination of workforce needs: LMICs note high shortages of specialized MH professionals, which in turn compromises the ability of systems to deliver comprehensive care [3, 6]. Third, many LMICs have not elaborated mental health policies. For instance, policies that promote the integration of mental health services with other sectors to ensure comprehensive approaches to both prevention of mental health problems and curative service provision are absent in many LMICs especially in Africa and South East Asia [3, 7].

Despite these challenges, scope for positive developments for LMICs and fragile contexts is evident. For example, primary and community level MH care are increasingly recognized as a cost-effective approach for addressing MH burdens; by empowering communities and close to community providers to address mental health problems increased prevention activities could take place and fewer specialist MH resources would be needed [8]. Transition to such approaches is slow, however, with inpatient care still dominating the MH care and support landscape, despite this being most relevant to severe – and thus a relatively small proportion of cases-only [8]. WHO data suggest that the weak connections between MH and other programmes within the health system as well as non-health sectors are a chief factor affecting this transition [8]. Although formal collaboration mechanisms between mental health care and primary health care (PHC) departments are present in many countries, there is still little active integration of mental health into PHC [8].

### Mental health in Lebanon

Limited evidence on the burden of mental health and psychosocial challenges is available for Lebanon. Studies among adults date back to 2003 and suggest a lifetime prevalence of any mental health disorder of 25.8 % [9]. Only a minority of the surveyed adults presenting with any mental health disorder were noted to have ever received professional treatment; even where this was the case, substantial delays (6 to 28 years) between the onset of disorders and onset of treatment were identified [9]. This treatment gap may be due to several factors. Recent studies focused on the help- and health-seeking behaviours of individuals suffering from mental and psychosocial issues highlighted that utilization of health services is shaped by high social stigma and treatment costs, limited trust in public health systems and concerns over compromised confidentiality [10–12].

The adequate provision of mental health services remains a major challenge in Lebanon. National expenditures on mental health are still far below needs [13]. The budget for Mental Health constitutes 5 % of the general health budget, and is mainly devoted to cover long stay inpatient cost in private hospitals [8]. Outpatient community based services are the responsibility of the private sector, however no budget is formally allocated from the Ministry for these services, except for provision of a small sub-set of medications for free [13]; community-based services such as day-treatment facilities are also lacking. Moreover, the health system still suffers from a shortage of mental health professionals despite increased needs for specialist care and a shortage of governmental hospitals able to cater to referrals [14].

Despite these challenges, a critical milestone for Lebanon’s mental health care sector was achieved in May 2014 with the launch of the National Mental Health Program (NMHP) within the Ministry of Public Health (MoPH), with the support of the World Health Organization (WHO), UNICEF, and International Medical Corps (IMC).One of the key initiatives of the program was to scale up availability of mental health care services via the implementation of the Mental Health Gap Action Programme (mhGAP) in primary health care centers within the MOPH network. The aim of the initiative was to reduce the evident mental health treatment gap, decrease stigma of mental health conditions, and build the capacity of health professionals [14]. Yet, due to budget cuts, support and supervision were placed on hold and the implementation of the initiative was interrupted.

### Rationale

Available estimates of the burden of mental health and psychosocial issues in Lebanon underscore the need for comprehensive mental health services. However, the health system has had limited capacity to scale up such services given financial and human resource shortages. Integration of mental health services into routine primary care may be one potential strategy implemented by the NMHP for enhancing service availability, similar to recommendations for other contexts [15]. However, little is known about the experiences and attitudes of currently active health care providers involved in mental health and psychosocial service provision at primary care level in Lebanon. Specifically, this cadre’s perceptions on their ability to address the mental health burden of beneficiary communities, and their perception on the health systems’ ability to support such service delivery, is unknown.

This study examines how health care providers offering mental health and psychosocial support services at primary care levels in two fragile regions of Lebanon perceive mental health and the health systems’ ability to address this burden, with a view to identify opportunities for strengthening mental health and psychosocial support service (MHPSS) implementation.

## Methods

### Research design and study setting

This study used a qualitative design to examine, from the perspective of care providers active at primary care levels, the factors and dynamics affecting onset of mental health challenges, associated health-seeking behaviors and ability of the health system to address the mental health burden. The study consisted of semi-structured interviews and group model building (GMB) workshops conducted in two contrasting settings in Lebanon: Beirut and Beqaa, and targeted health care providers involved in mental health and psychosocial service provision [16–18].

The two regions Beirut and Beqaa were purposively chosen to include settings with a diverse fragility profile [19]—i.e. settings experiencing a diverse set of overarching contextual stressors. Beirut is the capital city and the main urban center of the country and Beqaa is a largely rural region greatly impacted by the Syrian refugee influx. Beqaa region hosts the highest percentage of Syrian refugees in Lebanon, mostly residing in tented settlements and living in a weak civil society infrastructure, unlike Beirut which attracts the wealthier Syrian population and has relatively improved living conditions.

### Study population, sampling and recruitment

The study included a diverse set of health care providers (HCPs) who deliver care within each of the two selected settings.Participation was open to providers active in MHPSS service delivery for more than one year, including: nurses, clerks, and physicians working in private clinics, the public sector and/or in primary care facilities owned by the civil society. Staff of programs/projects offering psychosocial support in the two-targeted settings were also eligible for participation provided they have been active for more than 1 year.

A list prepared by the Ministry of Public Health (MOPH) was used to identify our study population in the selected two settings. The MoPH had completed a mapping exercise conducted by the National Mental Health Programme using the WHO “4Ws” mapping tool [20], and accordingly had identified all centers and staff who provide MHPSS services. Members of the research team contacted the HCPs through phone calls or site visits, explained the aims of the research study and solicited consent for an interview either on the same day or later of mutual convenience.

We recruited 36 HCPs active in mental health service provision from Beirut and Beqaa regions as presented in (Table 1). A total of 15 semi structured interviews were conducted with psychologists, nurses, social workers and general practitioners across genders. Further, 21 participants were recruited for the two GMB workshops that took place at the American University of Beirut (AUB).

**Table 1:** Participants characteristics by gender, specialty and setting

	Beirut (N = 21)	Beqaa (N = 15)
Semi structures interviews, N = 15		
Gender	Females = 5 Males = 4	Females = 4 Males = 2
Specialties	Nurses = 3 General Practitioners = 1 Psychologists = 3 Social workers = 2	Social workers = 3 Psychologists = 3
Group Model Building workshops N = 21		
Gender	Females: 9 Males 3	Females: 7 Males 2
Specialities	Nurses = 4 Social workers = 2 Pharmacists = 1 General Practitioner = 1 Psychologists = 2 Psychiatrists = 2	Nurses = 3 Social workers = 2 General Practitioner = 1 Psychologists = 3

### Semi-Structured interviews: data collection and analysis

We conducted 15 interviews with HCPs: nurses trained on mental health, social workers, general practitioners, psychologists and psychiatrists. All interviews were carried out in Arabic, audio-recorded, and lasted on average 20 min. The setting for interviews was decided based on the preference of the participant, with the majority of interviews being conducted at the health center. Interviews were semi-structured and included predetermined, open-ended questions on:

- HCP’s view on mental health and the causes leading to the onset of mental health issues.
- Professionals’ perceptions toward mental health problems.
- Fragility in the community and the health systems (factors affecting the patients’ access to care and factors affecting the provider’s ability to respond to the patients need)..

Interview recordings were transcribed verbatim, and the research team inductively coded all interviews and further engaged in thematic analysis using Dedoose [21]. Emerging themes were discussed in research group meetings and contrasted against the findings of the group model building workshops; differences between participant viewpoints based on gender, location and professional background were discussed and considered in the interpretation of findings.

### Group model building (GMB): data collection and analysis

In addition to interviews, two GMB workshops were organized: one workshop for HCPs active in the Beqaa’s (9 participants) and one workshop for HCPs active in Beirut (12 participants). Workshops were held at the American University of Beirut and transportation was arranged for participants to and from AUB. Group model building sessions are organized around a series of scripts which correspond to sequential activities that are carried out by participants with facilitation from the research team [22]. Scripts were elaborated by the research team, where relevant adapting scripts from Scriptopedia [23] or designing new exercises of relevance; a full list of scripts used is available in Additional file 1: Appendix S1.

Each GMB session consisted of three sections. First, participants were asked to draw rich pictures depicting the key factors that contribute or lead to the onset of mental health issues. Further, participants were asked to draw the key health seeking pathways of persons affected by a mental health issue, including drawings of challenges persons may face within the health system itself [24]. Second, participants were asked to develop graphs and describe trends depicting the mental health burden of the country, including changes in the prevalence of mental health cases, knowledge of mental health issues and awareness of the availability of MHPSS services among populations over time (1975–2018). Third, participants were invited to review the rich pictures and graphs developed and abstract a key set of variables relating to the onset of mental health issues, health seeking behaviours of communities, and challenges the health system faced over time in addressing mental health. Using these variables, researchers assisted participants in linking variables, prompting as relevant around causality, in order to create a preliminary causal loop diagram (CLD) depicting the dynamics of MHPSS onset, care seeking and associated care delivery. Finally, participants were prompted to identify particular points of fragility (i.e. areas of particular weakness) and also intervention (i.e. areas where intervention would be most strategic) in the depicted models.

### Analyses and framework

The CLDs generated by the participants during GMBs were transferred to Vensim [25]. Diagrams were iteratively refined using the notes taken during sessions and consolidated into one comprehensive CLD focused on outlining HCPs perceptions on factors contributing to the onset of MH challenges, community health seeking behaviours around MH and the health system’s ability to offer MHPSS services. This process was iterative and the research team proceeded stepwise. First, variable names and links between variables were reviewed and revised to reflect the causal logic expressed by participants in workshops. Second, comparative analyses were conducted examining points of commonality and difference between the CLDs developed by health care providers from the Beqaa vs. Beirut. Third, CLDs were consolidated (highlighting salient differences as appropriate) and feedback loops and critical pathways explaining the dynamics behind MH problems arising, care seeking and health system’s ability to offer MHPSS services were identified.

In a fourth step, these latter findings relating to the dynamics of the system were compared and contrasted with interview findings, and emerging insights were further contrasted against available literature. Inductive analyses of both interviews and GMBs as explained above identified both an appetite for the delivery of person-centered care (which HCPs noted as critical to successful MHPSS service delivery) and dynamics inhibiting the delivery of such services (with HCPs noting the system being geared to patient focused care). Thus, the research team retrospectively [26, 27] adopted a theoretical framing – focused on understanding differences between person vs. patient focused care as outlined in Starfield (2011) – to present findings [28]. Person-centred care extends the concept of patient-centred care and works towards close and direct relationships among individuals, communities and healthcare workers [29]. Whereas patient-centred care is commonly understood as focusing on the patient and disease—people-centred care encompasses these clinical encounters and includes attention to the health of people in their communities, the wider social determinants impacting upon health and the crucial role of persons and communities in shaping health services [30]. At minimum, implementation of person-centred care prioritizes people’s personal experiences of health and illness through hearing and respecting their perspectives and choices and incorporating their values and knowledge into the delivery of care.

### Triangulation of findings

This study is part of a larger project which aimed to examine the dynamics of mental health and psychosocial support from the perspective of both health care professionals and Syrian refugee and Lebanese host community members in Beirut and Beqaa, Lebanon. A detailed account of the methods, including analogous workshops and interviews with community members, and findings of said study can be found in Noubani et al. [10]. We comment on the triangulation of findings against those of this latter study throughout the presentation of results.

### Ethics

Ethical approval was obtained from the Institutional Review Board at the American University of Beirut (AUB) and Queen Margaret University (QMU).

### Reporting

The reporting of this study followed the consolidated Criteria for Reporting Qualitative Research (COREQ) Additional file 1: Appendix S2.

## Results

We first discuss how health providers perceive mental health issues and factors contributing to their onset as well as associated health seeking. Throughout, we compare providers accounts with those of community members, presented in a separate publication [10]. Further, we discuss the dynamics of the health system itself and challenges to MHPSS service delivery as perceived by health providers, highlighting challenges in the delivery of person-focused care as per Star field [28].

### Provider perceptions on factors affecting the onset of mental health conditions

**Fig. 1:**
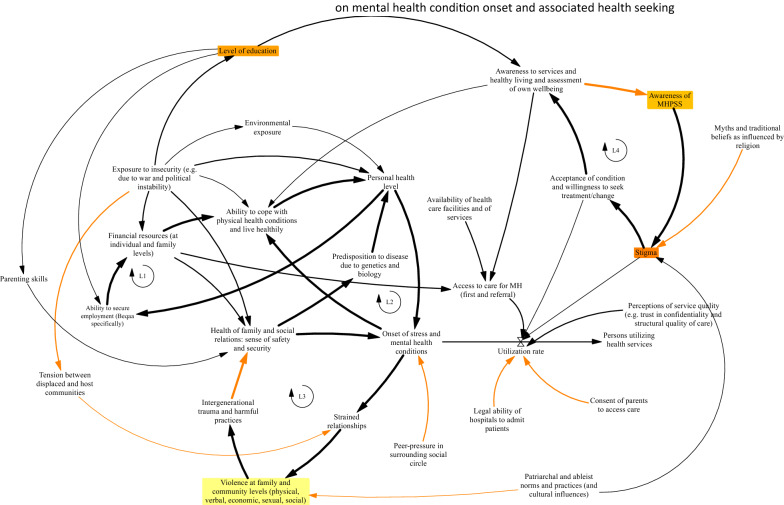
Causal loop diagrams describing provider perceptions on mental health condition onset and associated health seeking. Arrows in black describe accounts that were consistent between providers in Beirut and the Beqaa, arrows in orange refer to the Beqaa specifically. Variables in orange and yellow describe particularly fragile elements as identified by providers

In response to prompts on factors affecting the onset of stress and mental health issues, providers described complex and dynamic interactions, relating primarily to the populations’ (both Lebanese and Syrian) exposure to war and political instability, a tense community and family environment, and precarious financial conditions within Lebanon. Figure 1 presents a CLD illustrating a consolidated account of the factors precipitating compromised wellbeing and MH condition onset, and associated health seeking.

Overall, we identify three main feedback loops from provider’s accounts. Loop L1 (see left of diagram, Fig. 1), outlines how the level of resources available at family levels directly links to a persons’ ability to secure and take care of their physical health, and over time, their ability to therefore secure employment and contribute to their family’s resources. Providers noted that particularly in the Beqaa, where there were limited employment opportunities and where tensions between local host and Syrian refugee communities were high, resources at household levels were scarce and over time physical health suffered. Health providers linked the availability of employment opportunities further to the political instability in Lebanon and the wars witnessed in the region, noting these factors were main causes for the impairment of economic growth. The financial constraints in the country left people unable to secure their livelihood and afford to meet family needs such as school tuition fees and accessing healthcare services.

*“Pressure mainly stems from the situation in the country. Schools are expensive, there is no work, and hospitals are expensive and inaccessible. If a person gets sick, he has to pay huge sums of money for hospitalization if he does not have insurance or social security.” A nurse living in Beirut*.

Moreover, providers talked about poverty and limited employment as major challenges in the country and a main contributor to mental health issues.

*“Poverty is a big issue that carries with it depression.” A HCP from Beirut*

Further, providers also linked exposure to wars and displacement to the health of families and communities as well as physical health issues. Providers noted that potential trauma or high levels of distress due to the loss of loved ones could directly compromise a person’s health or their ability to look after their health, or over time may lead to epigenetic changes impacting the health of future generations (see Loop 2, center of Fig. 1).

*“In refugee communities, due to the war, some people face psychological shocks that cause imbalance.” HCP from Beqaa*

Providers also noted that displacement itself often prompts the emergence of strained community relationships. Clashes between diverse cultures, for example, may compromise community mental health.

*“People who immigrated because of the war might have experienced a change in culture and traditions which affected their mental health.” HCP from Beqaa*

Additionally, HCPs from the Beqaa region specifically highlighted how community relations and strained relationships could lead to violence occurring at community and family levels (see Loop 3, lower left Fig. 1). Participants from this group spoke about how the cultural norms and patriarchal system in the Beqaa region shaped both ableist norms and masculinity identities within which able bodied individuals are advantaged and within which men need to hold power and dominate in roles within the family and the community. When such roles are threatened interpersonal violence often ensues, and providers noted that exposure to violence over time may also lead to intergenerational trauma taking root, thus affecting family wellbeing further, and leading to the onset of chronic stress and mental health issues for whole families. Such accounts are very similar to those noted by community members [7], particularly within the Beqaa.

Accounts of what prompts the onset of chronic stress and mental health conditions were largely consistent across providers from the Beqaa and Beirut areas. The main differences highlighted relate to increased tensions between communities in the Beqaa and providers impressions of increased interpersonal violence and intergenerational trauma within this region.

As outlined above, the information provided by health care providers was overarchingly consistent with that of community members [10]. However, in contrast to communities, providers reflected more on how genetic predisposition and the ability of persons to actively care for one’s physical health can affect stress and wellbeing levels (see Loops L2).

## Provider perceptions of community health seeking behaviours

Providers in both settings reflected on factors that shape community level health seeking behaviours (see centre of Fig. 1, utilization rate variable referring to utilization of formal health services). Social stigma was noted as the main barrier to access and utilization of mental health services. Participants noted that stigma affects both the acceptance of mental health conditions and willingness of the affected person or patient to seek or continue seeking treatment for improvement. Stigma was noted to be fueled by lack of knowledge and limited awareness of mental health as well as myths and traditional beliefs as influenced by religion (see Loop L4, right top hand side of Fig. 1).

Healthcare providers clarified that stigma was present in communities and in the health system itself as well; patients for example may fear the judgmental attitudes of providers towards mental health and therefore not seek care. Perceptions of communities, as related to trust in providers and expectations of providers keeping problems confidential, and perceptions on the structural quality of care offered at health facilities, were noted to affect health seeking. (see right hand side of Fig. 1).

Additionally, providers noted that it may be unclear where exactly in the health system community members should seek help for mental health conditions. Providers noted that general practitioners were generally approached for physical symptoms, however the latter may turn out to be related to psychological challenges.

Similar to community member accounts [7], providers mentioned that people usually seek support from their friends, families, and religious figures prior to seeking support from the health system.

*“HP: Most people first seek friends, people they rely on or trust. The second step when they don’t have much awareness about mental health, they might seek GPs because the symptoms are portrayed physically. It could be a stomach pain or headache. Your mental health issues might have been around for so long that they have compromised your immune system.” HCP from Beirut*

*“In simple cases like anxiety and depression, they might resort to their family.“ HCP from Beqaa*

*“HP: Many people who suffer from bipolar disorder and schizophrenia are taken to religious figures instead especially the Syrians coming from traditional villages.” HCP from Beqaa*

Providers clarified that the community view utilization of formal mental health services as the last resort, and further highlighted the limited availability and affordability, as well as perceptions of low service quality, as main barriers of access to mental health services. These accounts were consistent with those of community members, across both the Greater Beirut area and the Beqaa [10].

### Systems barriers to person focused care

Provider accounts from the GMB workshops and interviews were concordant and suggestive of a health system geared towards addressing issues of acute care (principally around physical conditions) rather than chronic mental health needs. We contrast emerging themes to the person-focused care framework [29] and identify themes which describe barriers or facilitators to putting in place person-focused mental health services in Lebanon.

Across GMBs providers offered relatively brief accounts of the health system, however highlighted the low resource base in relation to mental health (specifically trained human resources) and the need to integrate mental health service delivery in routine primary health care so as to ensure continued care for affected persons. Drawing principally on interviews, where providers were able to express views at greater length, we identify three main themes which illustrate how the Lebanese health system is not yet ready, however willing, to offer person-focused care [29].

#### Barrier 1: Lack of cohesive and coordinated health management information system compromises quality of care

Providers mentioned that no harmonized filing or health information system exists within the current Lebanese system. Thus, the full medical or psychological history of persons needs to be elicited at each patient encounter, potentially compromising the wellbeing of the patient further or sparking anger, particularly in case of mental health conditions.

*“The administrative process and structure plays a role. The patient repeats the things to the nurse, then GP, then they have to go to different floors. We should be aware of these things to facilitate the process for the patient. The easier, the better. What if the patient has no energy to repeat the same story again?” HCP from Beirut*.

Providers reflected that the system is currently not built for mental health conditions and that lack of a cohesive and coordinated system, across diverse providers, severely limits file sharing and also referral and follow-up.

*“Our problem is with harmonizing files of patients and data management. This is our biggest problem, and we brought it up with the Ministry. We cannot solve it now. Its solution is a bit far. We are five NGOs. Each has its system and tools. The data is not harmonized. We don’t even have online data.” HCP from Beirut*.

#### Barrier 2: Limited human resource capacity for mental health due to limited training and scarce resources

Providers mentioned that the shortage in human resources specialized in mental health is a major challenge, especially in the Beqaa area. Specialized cadres are scarce within the country, and when available largely focused around the capital.

*“Another thing is that there should be an analysis to find out which areas in Lebanon lack psychotherapists. As I mentioned before, the further you go outside Beirut, the less psychotherapists you’ll find. So, there should be a needs analysis to know in which regions we should hire more psychotherapists.” HCP from Beqaa*

Providers noted that among generalist staff, limited training on mental health and psychosocial support had been provided, and where training had been provided – as for example was the case with MhGAP training during 2015–2016, this was largely around how to screen, manage and refer cases when needed, with limited refresher or follow-up programs further taking place. Participants related the lack of training to general funding limitations experienced by the Ministry.

*“Lack of training also related to the lack of funding to the ministry. GPs were trained to screen for mental health issues and refer to mental health specialist when necessary. Yet refresher training are no longer being done “. HCP from Beqaa*

Participants explained that given the limited training on case management, health providers lack the skills and confidence needed to admit and treat severe cases. In the Beqaa area, admission of severe cases may be refused given lack of capacity.

*“In the Bekaa for example, if someone needs hospitalization, we do not have the hospitals that would accept these cases. Even if the mental health patient gets admitted to a hospital, there is concern about some extreme cases. Sometimes the hospital would refuse a patient because they are scared the patient might jump out of the window for example or hurt himself. They are not trained to accept these cases.” HCP from Beqaa*

Participants also noted that training for staff should be comprehensive, and additionally tackle the high levels of stigma prevalent even within the health service. Some providers for example consider mental health issues as a taboo.

*“Staff inside the hospital have a stigma and say this is a ward for crazy people. We received this feedback, and we immediately launched a sensitization plan. Mental health staff carry out sensitization sessions rather than trainings to sensitize the hospital staff and front liners about the definition of mental health and the Community Mental Health Center and it services” HCP from Beqaa*

#### Barrier 3: Limited service integration and coordination compromises the systems’ ability to address the mental health burden

Providers acknowledged multi-factorial determinants of mental health issues and described the need for interventions that aim for prevention rather than management. The health system relies on general practitioners referring patients to mental health specialists, however ambiguity in case management remain, with limited clarity over focal points for specific cases.

*“Focal points are not clear, within the components we tackled. The communication path between all components should be clear to facilitate the pathway for the patient. If somebody is absent, who is the replacement? All these are important matters. If the case manager is absent and the patient comes, and I have no clue about the patient’s file, how can I help them?” HCP from Beirut*.

Further, particularly where mental and physical health issues cannot be easily disentangled, responsibilities for care and service coordination and integration remain a challenge.

*“I think it is important for each hospital to have a mental health department, especially in the Bekaa hospitals or remote areas. Sometimes, for instance, in cases of bedwetting among children, we need to know if the case is medical or if the child is being harassed. If I refer a child who is being harassed to a pediatrician who is not aware of harassment and mental health issues, he might not be able to help the child and might drag him into a spiral of medication and examinations.” HCP from Beqaa*.

Despite the above-mentioned, participant accounts also clearly highlight the readiness of health providers to engage more fully with the delivery of person-focused and integrated mental health services.

#### Facilitator 1: Providers perceive mental and physical health as interrelated phenomena to be treated coherently and in conjunction with each other

Providers acknowledged that communities and other providers with limited mental health training often focus on physical symptoms and illness, but in our accounts, providers reflected on these being interrelated phenomena. They clarified that physicians are trained to take into consideration mental health issues when there is no clinical reason for the physical pain of the patient.

*“HP: I can imagine that screening is very essential for GPs. After GPs run their tests, and they notice there is no physical cause, the GP would know that there is a mental health issue” HCP from Beqaa*.

Participants further reflected that mental health issues are due to multiple factors that are related to socioeconomics, family and social relations, genetics and adopted lifestyles (as mentioned in the previous section on factors leading to the onset of mental health issues).

#### Facilitator 2: Providers are ready to build trusting and open relationships with patients

HCPs talked about the importance of their role in building trust with the patient and helping them to be open about his/ her mental health issues in a secure and safe environment. Providers emphasised that patients still view mental health issues as a taboo, which is why continued open communication is important.

*The trust building process is key because for some patients it is taboo, they don’t like to talk about it” HCP Beirut*.

*“The first thing we used to work on is discussing their needs and building trust. Then, we would get to the phase where they would set targets they want to improve.” HP Beqaa*.

However, providers acknowledged that building trust remains a challenge given both high workloads and ambiguity around care coordination. Additionally, HCPs mentioned that most patients still consider mental health as a taboo and that physicians themselves may find it difficult to open up the topic with the patient for the first time.

#### Facilitator 3: Providers are concerned with the evolution of people’s experienced health problems

Providers noted that working with other professionals to tackle not just health issues but wider social issues leading to the emergence or exacerbation of mental health issues is crucial. For example, they clarified that social workers may be more experienced in building trust with communities and helping them open up and communicate about their mental health concerns.

Providers also mentioned the need to organize outreach campaigns in cooperation with different civil organizations and primary healthcare centers as well as the need to seek support of the social media to spread awareness among the community. They noted that the challenge with current outreach activities is that they are targeted primarily at Syrian refugees, leaving a gap for Lebanese persons who prefer seeking care from private clinics.

*“There is a lot more we could do. We could run outreach campaigns in cooperation with different civil organizations and we could have better presence on social media.” HCP from Beirut*

*“The outreach affects Syrians more than the Lebanese. As for the Lebanese, we usually reach them through healthcare centers, but it is not effective since the Lebanese tend to go to a doctor or a private clinic instead.” HCP from Beqaa*

### Points of fragility and suggested interventions

GMB participants across the Beqaa and Beirut were asked to reflect on the causal loop diagram they developed (Fig. 1 and Additional file 1: Appendix S1) and to vote for the top weaknesses and issues affecting mental health service utilization and delivery in Lebanon. HCPs across both settings identified the issue of stigma which is fueled by lack of awareness to MH among the population including the potentially stigmatizing attitudes of the HCPs themselves towards mental health. Additionally, HCPs reflected on the limited availability of services due to the limited funds and human resources within the country, which affects the continuous training and education of the providers and even availability of medications. HCPs from the Beqaa additionally noted the issue of violence at the family and community levels as a major factor leading to mental health issues among the population in Beqaa.

At the end workshops, HCPs were given the chance to suggest potential interventions to improve the situation of mental health service delivery in Lebanon (see Table 2). Across accounts, we note staff focusing on factors which may undermine wellbeing and/or undermine the health systems’ ability to deliver care. Conducting awareness campaigns was a common suggestion to fight stigma, stop inter-personal violence and help people seek mental health care. Providers from the Beqaa recommended the need for multisectoral coordination and collaboration among the different ministries, taskforces and field practitioners, including the need to secure funds and financial resources for this care delivery. Beirut HCPs instead suggested the need for more research that assess the burden of mental-ill health, current awareness levels and gaps in service provision; they noted this could be used for advocacy purposes to support raising funds (Table 2).

**Table 2:** Suggestions and recommendations by the providers

HCPs from Beirut	HCPs from Bekaa
1. Strengthening public awareness and engagement of people having mental health diseases to improve their knowledge and awareness to available services and reduce social stigma within the community and among healthcare providers. 2. Enhancing investments for mental health and budget allocations to train healthcare providers. 3. Innovation and implementation should be guided by research 4. Conducting research and generating evidence on the burden of mental health disorders and the status of health service provision.	1. Strengthening awareness campaigns against gender based violence and abuse 2. Empowering Multisectorial collaborations to promote and protect the mental wellbeing. 3. Improving the responsiveness of the legal system 4. Strengthening investments in mental health training programs: training security forces and medical staff on how to deal with mental health patients especially the severe cases

## Discussion

The CLD model developed collaboratively between HCPs gave great insights into the factors contributing to the onset of mental health issues and associated health seeking behaviours among community members. The CLD established the perceived causal relationships that explain factors leading to onset of stress and mental-ill health, and identified points of leverage for intervention. It is worth underscoring the bidirectional association between physical health and mental health, as explained by the HCPs, and its impact on patients’ quality of life. War experiences and socioeconomic stressors compromise physical health or the ability to actively care for it, which in return aggravate the mental and psychological ill-health of the affected person influencing his/her social and family relations as well as his/her health and wellbeing outcomes. The identified factors perceived by HCPs were overarchingly consistent with those acknowledged by community members [10]. These elements were mainly linked to experiences of war and displacement, as well as integration challenges between the Lebanese host community and Syrian refugees, further exacerbated by financial constraints and limited employment opportunities. Additionally, the CLD model helped providers reflect on the role of cultural norms and patriarchal systems that shape community and family relations, especially in the Beqaa region, potentially highlighting the role of the latter in prompting intergenerational trauma and violent practices. Elaborated health seeking pathways suggest that the main barriers for seeking help and influencing the patient- provider relationship relate to stigma—both within the community and health system itself. HCPs clarified that seeking professional care is normally considered the last resort for improving mental wellbeing due to the lack of awareness to available MHPSS services, financial constraints and lack of trust in service quality. This finding points to the need for strengthening community and primary care based services specifically also around tackling stigma in local communities and offering early detection and referral.

The study offers an account of the current shape and design of the Lebanese health system, geared towards addressing acute and chronic physical conditions, but potentially ill-equipped for managing mental health conditions. The overall architecture of the health system focuses on providing patient centered rather than person focused care, and therefore, offer limited attention and support for meeting the non-physical needs of individuals. Challenges within the system include the lack of a coordinated health information system, limited service integration and coordination across diverse providers, including as/when physical and mental health challenges converge, in addition to a generically low resource base in the country resulting in limited trained human resources. However, HCPs accounts illustrated the willingness of health professionals to engage with integrated person- focused care models; emphasis was placed on offering both in-service and community level support from multiple sources, spanning diverse sectors, including social services and education.

Results presented here echo those outlined in a situation analysis of mental health services in Lebanon from 2009 [11]. Previously noted barriers included shortages in mental health experts, lack of funding to train health professionals in managing mental health cases, in addition to an acknowledgment of limited time to treat mental health disorders at the primary care level [11, 30]. Studies also reported that social stigma among both patients and providers was a major barrier for optimal delivery of mental health care, as well as health literacy among those who suffer from a mental health condition [10, 11, 31].

While the barriers identified in 2009 still persist, our study also illustrates that further positive developments had taken place but were interrupted. For example, the NMHP set out a strategic plan that aimed to ensure the development of a sustainable mental health system that guarantees the provision and universal access to preventive and curative mental health services. This was partially achieved through delivery of mental health trainings (on mhGAP specifically) at primary care level and attempting scale up of trainings and delivery of basic services across the primary care network. While some providers benefited from this training and therefore now demonstrate both increased skills and willingness to deliver MHPSS services, the initiative was interrupted as a result of funding shortages and additional frustrations arose as the wider systems needed to deliver such services appropriately were not in place. For example, shared patient record systems, as highlighted by our participants, would be one step towards strengthening the system’s ability to better address or refer mental health cases.

The challenges identified in the current study are similar to those reported by the Lancet commission on global mental health and sustainable development [32]. The commission recommended a reframed global mental health agenda that aims to reduce the contribution of mental health to the global burden of disease through the adoption of several strategies [32]. Foremost, integrating mental health care within universal health coverage would ensure that the concept of indivisibility of physical and mental health is operationalized [32]. The commission also emphasized the need for strengthening public awareness and engagement to reduce stigma and discrimination within communities and stressed the need for enhancing budget allocations for mental health [32].

### Strengths

Our study provides an up to date understanding of the health system’s ability to address the burden of mental health in Lebanon and at the primary care level in particular.Points for intervention in the system were identified by health professionals directly involved with care delivery; we therefore offer their suggestions for policy makers to consider in relation to strengthening mental health systems in Lebanon.

### Limitations

We faced major challenges when identifying and recruiting participants for the study. The small sample size (n = 36) reflects the limited number of mental health specialists at the primary care, especially in the Bekaa region. The limited number of participants in GMBs may also have limited emergence of rich data. Additionally, not all invited HCPs were able to attend our workshop since it was challenging for health professionals to commit to full day attendance, especially for those who reside in the Beqaa region and needed to commute to Beirut.

## Conclusions

Mental illness is a major public health problem with implications at the individual and national levels in Lebanon. A concrete and coordinated effort, and further investments, are required to address the burden of mental ill-health in Lebanon. This paper highlights the willingness and potential for Multisectorial action for strengthening community care and primary care according to person-centred care principles.

## Supplementary Information

**Additional file 1. ** Appendices.

## Data Availability

All data generated or analyzed during this study are included in this published article [and its.

## Abbreviations

DALY: Disability adjusted life years
LMICs: Low and middle-income countries
NMHP: National Mental Health Program
MoPH: Ministry of Public Health
WHO: World Health Organization
MhGAP: Mental Health Gap Action Programme
MHPSS: Mental health and psychosocial support
HCPs: Health care providers
GMB: Group Model Building
CLD: Casual Loop Diagram
MH: Mental Health
