# Supplementary material for: Readiness to deliver person‐focused care in a fragile situation: the case of Mental Health Services in Lebanon
Source: Int J Ment Health Syst. 2021 Mar 2;15:21. doi: 10.1186/s13033-021-00446-2 (PMC7923303; doi:10.1186/s13033-021-00446-2)
Supplement: Supplementary file 1 — Additional file 1. Appendices. [file 13033_2021_446_MOESM1_ESM.docx]

**Appendix 1**

**Script 1: Health providers GMB**

| **Time** | **Activity** |
| --- | --- |
| **(min)** |  |
|  |  |
| 10 | Welcome and project introduction |
|  |  |
| 10 | Demonstrating use of GMB methods |
|  |  |
| 30 | **Rich pictures** |
|  | Ask participants to draw a typical person with MHPSS condition (5-10 minutes). What condition are |
|  | you drawing? |
|  | What are the causes for these conditions/issues that prompt condition development? (5-10 minutes) |
|  | Can you draw a journey around these patients? Fill in the drawing with a pre-illness drawing – what |
|  | were these patients like/doing before/as they arrive to clinic? What is their continued journey likely to |
|  | be? (10-15 minutes) |
|  | Now draw the providers and health care workers surrounding the patient – where are they present? |
|  | What are their roles? |
|  |  |
| 20 | **Reference modes** |
|  | Participants will be asked to draw graphs representing the last 10 years. Prevalence of MHPSS |
|  | complaints, prevalence of MH conditions, knowledge of all conditions among the providers present. |
|  |  |
| 15 | **Break** |
|  | Modelling team gathers all materials and elicits/selects a first set of variables to base next activity on. |
|  |  |
| 25 | **Variable elicitation** |
|  | Ask participants to split into groups according to their age group. For each of the groups, ask them to |
|  | use sticky notes and brainstorm: issues contributing to the onset and exacerbation of each disease, |
|  | factors affecting the patient journey (from when patient arrives at clinic and then returns home/back |
|  | to clinic etc), factors affecting a provider’s ability to respond to patient needs (15 minutes) |
|  | Comparison of variables (10 minutes): If/where variables coincide, propose a common ‘framing’ of |
|  | variables be used. Note the diverging variables though allow each group to continue using these as |
|  | needed going forward. |
|  |  |
| 15 | **Inter-relationship diagram** |
|  | Place the sticky notes of variables in a circle and encourage participants to think about which variables |
|  | influence one another. Draw the links where influences are noted. |
|  |  |
| 60-70 | **Causal loop diagram development (Part 1)** |
|  | Within each of the groups, the modelling team provides participants with a seed model depicting a |
|  | patient journey (to be developed from interviews) asking them to introduce the variables previously |
|  | elicited within this. |
|  | Prompt the groups to consider where and how the health system – i.e. each of them as providers – |
|  | impacts on the journey. Start with adding in variables around human resources (e.g. where does the |

community health worker go? What do they do?) And encourage participants to expand upon the model as much as possible.

Once models are elaborated (approx. 45 minutes), groups will be encouraged to feed back to one another. The possibility of elaborating a single comprehensive model will be explored and facilitators will be interrogating the causal nature of links each time a new link is proposed.

1. **Lunch**

60-70 **Causal loop diagram development (Part 2)**

Using models elaborated within the first half of the day, participants will now be asked to work together to merge models – as services are offered within the same spaces and clinics, providers will be encouraged to think of care integration.

Prompt the group to consider issues of resource use, potential guidelines that may come in to assist care delivery, training etc.

1. **Identifying links or points of ‘fragility’**

The modelling team will prompt participants to identify ‘fragile’ links or points of weakness. Preliminary interviews within Sierra Leone suggest that fragility arises both due to hard- (e.g. limited medicines) and soft-ware (e.g. limited skill training) constraints. The modelling team will encourage participants to identify these within a diagram and potentially rank these using a card sorting task.

Once points of ‘fragility’ are identified, the modelling team will prompt participants to identify various solutions/interventions with potential to improve the situation.

**Appendix 2: Consolidated criteria for reporting qualitative studies (COREQ): 32-item checklist**

Developed from:

Tong A, Sainsbury P, Craig J. Consolidated criteria for reporting qualitative research (COREQ): a 32-item checklist for interviews and focus groups. *International Journal for Quality in Health Care*. 2007. Volume 19, Number 6: pp. 349 – 357

| **No. Item** | **Guide questions/description** | **Reported on Page #** |
| --- | --- | --- |
| **Domain 1: Research team and reﬂexivity** |  |  |
| *Personal Characteristics* |  |  |
| 1. Inter viewer/facilitator | Which author/s conducted the interview or focus group? |  |
| 2. Credentials | What were the researcher’s credentials? E.g. PhD, MD |  |
| 3. Occupation | What was their occupation at the time of the study? |  |
| 4. Gender | Was the researcher male or female? |  |
| 5. Experience and training | What experience or training did the researcher have? |  |
| *Relationship with participants* |  |  |
| 6. Relationship established | Was a relationship established prior to study commencement? |  |
| 7. Participant knowledge of the interviewer | What did the participants know about the researcher? e.g. personal goals, reasons for doing the research |  |
| 8. Interviewer characteristics | What characteristics were reported about the inter viewer/facilitator? e.g. Bias, assumptions, reasons and interests in the research topic |  |
| **Domain 2: study design** |  |  |
| *Theoretical framework* |  |  |
| 9. Methodological orientation and Theory | What methodological orientation was stated to underpin the study? e.g. grounded theory, discourse analysis, ethnography, phenomenology, content analysis |  |
| *Participant selection* |  |  |
| 10. Sampling | How were participants selected? e.g. purposive, convenience, consecutive, snowball |  |
| 11. Method of approach | How were participants approached? e.g. face-to-face, telephone, mail, email |  |
| 12. Sample size | How many participants were in the study? |  |
| 13. Non-participation | How many people refused to participate or dropped out? Reasons? |  |
| *Setting* |  |  |
| 14. Setting of data collection | Where was the data collected? e.g. home, clinic, workplace |  |
| 15. Presence of non-participants | Was anyone else present besides the participants and researchers? |  |
| 16. Description of sample | What are the important characteristics of the sample? e.g. demographic data, date |  |
| *Data collection* |  |  |
| 17. Interview guide | Were questions, prompts, guides provided by the authors? Was it pilot tested? |  |
| 18. Repeat interviews | Were repeat inter views carried out? If yes, how many? |  |
| 19. Audio/visual recording | Did the research use audio or visual recording to collect the data? |  |
| 20. Field notes | Were ﬁeld notes made during and/or after the interview or focus group? |  |
| 21. Duration | What was the duration of the inter views or focus group? |  |
| 22. Data saturation | Was data saturation discussed? |  |
| 23. Transcripts returned | Were transcripts returned to participants for comment and/or correction? |  |
| **Domain 3: analysis and ﬁndings** |  |  |
| *Data analysis* |  |  |
| 24. Number of data coders | How many data coders coded the data? |  |
| 25. Description of the coding tree | Did authors provide a description of the coding tree? |  |
| 26. Derivation of themes | Were themes identiﬁed in advance or derived from the data? |  |
| 27. Software | What software, if applicable, was used to manage the data? |  |
| 28. Participant checking | Did participants provide feedback on the ﬁndings? |  |
| *Reporting* |  |  |
| 29. Quotations presented | Were participant quotations presented to illustrate the themes/ﬁndings? Was each quotation identiﬁed? e.g. participant number |  |
| 30. Data and ﬁndings consistent | Was there consistency between the data presented and the ﬁndings? |  |
| 31. Clarity of major themes | Were major themes clearly presented in the ﬁndings? |  |
| 32. Clarity of minor themes | Is there a description of diverse cases or discussion of minor themes? |  |
